# Dengue Outbreak in Mombasa City, Kenya, 2013–2014: Entomologic Investigations

**DOI:** 10.1371/journal.pntd.0004981

**Published:** 2016-10-26

**Authors:** Joel Lutomiah, Roberto Barrera, Albina Makio, James Mutisya, Hellen Koka, Samuel Owaka, Edith Koskei, Albert Nyunja, Fredrick Eyase, Rodney Coldren, Rosemary Sang

**Affiliations:** 1 Arbovirus/Viral Hemorrhagic Fever Laboratory, Center for Virus Research, Kenya Medical Research Institute (KEMRI), Nairobi, Kenya; 2 Entomology and Ecology Activity, Dengue Branch, Centers for Disease Control and Prevention, San Juan, Puerto Rico; 3 United States Army Medical Research Directorate—Kenya (USAMRD-K), Nairobi, Kenya; Mahidol University, THAILAND

## Abstract

Dengue outbreaks were first reported in East Africa in the late 1970s to early 1980s including the 1982 outbreak on the Kenyan coast. In 2011, dengue outbreaks occurred in Mandera in northern Kenya and subsequently in Mombasa city along the Kenyan coast in 2013–2014. Following laboratory confirmation of dengue fever cases, an entomologic investigation was conducted to establish the mosquito species, and densities, causing the outbreak. Affected parts of the city were identified with the help of public health officials. Adult *Ae*. *aegypti* mosquitoes were collected using various tools, processed and screened for dengue virus (DENV) by cell culture and RT-PCR. All containers in every accessible house and compound within affected suburbs were inspected for immatures. A total of 2,065 *Ae*. *aegypti* adults were collected and 192 houses and 1,676 containers inspected. An overall house index of 22%, container index, 31.0% (indoor = 19; outdoor = 43) and Breteau index, 270.1, were observed, suggesting that the risk of dengue transmission was high. Overall, jerry cans were the most productive containers (18%), followed by drums (17%), buckets (16%), tires (14%) and tanks (10%). However, each site had specific most-productive container-types such as tanks (17%) in Kizingo; Drums in Nyali (30%) and Changamwe (33%), plastic basins (35%) in Nyali-B and plastic buckets (81%) in Ganjoni. We recommend that for effective control of the dengue vector in Mombasa city, all container types would be targeted. Measures would include proper covering of water storage containers and eliminating discarded containers outdoors through a public participatory environmental clean-up exercise. Providing reliable piped water to all households would minimize the need for water storage and reduce aquatic habitats. Isolation of DENV from male *Ae*. *aegypti* mosquitoes is a first observation in Kenya and provides further evidence that transovarial transmission may have a role in DENV circulation and/or maintenance in the environment.

## Author Summary

The first dengue outbreak in Kenya was reported in 1982 in the coastal region. This was followed almost 30 years later by the 2011 dengue outbreak in Mandera, northern Kenya and subsequently in Mombasa city in the coastal region (2013–2014). An entomologic investigation was conducted to establish the density of mosquito species causing the outbreak. Affected parts of Mombasa city were identified with the help of public health officials. Adult mosquitoes were collected using various tools, processed and screened for dengue virus. All indoor and outdoor containers in every accessible house and compound within affected suburbs were inspected for *Ae*. *aegypti* immatures. Over 2,000 adult *Ae*. *aegypti* mosquitoes were collected and 192 houses and 1,676 containers inspected for *Ae*. *aegypti* immatures. Although jerry cans (18%) and drums (17%) were the most productive, there was site-specificity in container type productivity. Therefore all containers would be targeted for effective control of the dengue vector, including proper covering of water storage containers and eliminating all discarded outdoor containers through a public environmental clean-up exercise. However, providing reliable piped water to all households in Mombasa city would be a long-term solution to reduce the risk of dengue transmission.

## Introduction

Dengue virus (DENV) is a member of the genus flavivirus (family *Flaviviridae*) that is transmitted principally by *Aedes aegypti* mosquitoes in an *Ae*. *aegypti*-human cycle [1], sometimes resulting in epidemics. Although the presence of other *Stegomyia* spp. including *Ae*. *simpsoni* complex, *Ae*. *africanus* and *Ae*. *vittatus* in disease endemic areas in Kenya in sympatric/allopatric manner with *Ae*. *aegypti* have been documented [2], their role in DENV transmission remains unknown. *Ae*. *aegypti* is well distributed in the tropical and subtropical regions and readily develops in water held in artificial, often man-made, containers in and around human habitations [1], hence it is well adapted to domestic and urban settings [3]. The vectors live so close to humans on whom they preferentially feed, and DENV transmission can occur even when *Ae*. *aegypti* population densities are low [4,5]. The other known vector of DENV, *Ae*. *albopictus*, is not as associated with humans or their habitats as *Ae*. *aegypti*, and is responsible for dengue transmission mainly in Asia [6]. Whereas *Ae*. *albopictus* has recently been documented in parts of Central and West Africa including Equatorial Guinea, Cameroon, Gabon and Mozambique [7,8,9], surveillance conducted from 2007–2011 did not detect occurrence of *Ae*. *albopictus* in the coastal sites [2].

DENV causes dengue fever (DF), an acute mosquito-borne viral infection. DF is presently the world’s most important re-emerging arboviral disease with over 50% of the world’s population at risk of the disease and 50% residing in dengue endemic countries [10]. Approximately 3.6 billion people are currently at risk of dengue infection in over 100 countries of Asia, Americas and Africa [11]. It has been estimated that 390 million dengue infections occur worldwide annually [12].

The epidemiology and public health effect of dengue in Africa is poorly understood, although the vectors of DENV are widely distributed [13]. Dengue diagnosis is likely confounded by other diseases such as malaria and lack of laboratory diagnostic capability [14,15]. For example in regions endemic for malaria, 70% of febrile illnesses are treated as presumptive malaria or designated as having fever of unknown origin, hence the potential for misdiagnosing dengue as malaria. The distribution of dengue vectors and several other factors including rapid population growth, unplanned urbanization, and increased international travel increase the risk of dengue transmission [16]. Indeed, over the past 5 decades, dengue cases have been reported in many countries in sub-Saharan Africa [10] including European travelers returning from Tanzania, Zanzibar, the Comoros, Benin, Cape Verde, Gunea Bisau and Senegal [17–20] and the 2011–2013 outbreaks in Angola and Kenya [21,22]. This apparent emergence of DENV in most of Africa might be due to increased awareness of the disease, availability of better diagnostic tests, and improved access to specialized laboratory facilities [23].

Although Kokernot et al suggest that dengue existed in Africa as far back as 1926 [24], the first outbreak in eastern Africa was in Comoros in 1948 and later in 1983 and 1984 [25]. Between 1977 and 1979, a major outbreak caused by dengue 2 was reported in the Seychelles Islands affecting >75% of the population [26]. The Seychelles outbreak was followed by the first outbreak of DF, caused by dengue 2 virus, in Kenya along the coast in 1982 [27]. In 2004, DENV IgG antibodies were detected among humans in Malindi [28] suggesting continued circulation of this virus on the coast of Kenya. In 2007, a dengue 2 outbreak was reported in Gabon [29]. Also during this year, DENV antibodies were detected in 7 of 8 of the previous administrative provinces of Kenya (all except Nairobi) [30]. More recently in 2014, a dengue outbreak occurred in the United Republic of Tanzania [31], while between November 2011 and February 2014, an outbreak involving three DENV serotypes (1, 2 and 3) occurred in Mandera in northern Kenya [32] and in Mombasa city located on the coast of Kenya, where 58% of the suspected hospital cases (n = 267) were positive for dengue infection by RT-PCR [22]. Based on this data, we initiated entomologic surveillance activities to establish the mosquito species associated with the dengue cases, determine the densities of immature stages of the mosquitoes, identify the most productive container types in areas with ongoing DENV transmission and estimate the density of adult *Ae*. *aegypti* mosquitoes inside and around houses in areas with dengue cases. Data generated would lead to recommendations on control measures aimed at reducing the *Ae*. *aegypti* population densities [33] to stop further transmission.

Because dengue infection rates in *Ae*. *aegypti* are typically low [5] to base a surveillance and risk assessment program on entomological infection rates (EIR), this entomologic investigation was based largely on larval indices (i.e. container index (CI), house index (HI) and Breteau index (BI)). The Pan American Health Organization (PAHO) and World Health Organization (WHO) have described threshold levels for dengue transmission as low HI<0.1%, medium HI 0.1%–5% and high HI>5%. However, there is weak association between these indices and DENV transmission [34,35], hence they are limited to indicating vector presence or absence [36]. Because these threshold indices also differ from place to place [37], recent studies have recommended an area-specific re-evaluation of the utility of larval indices [38].

## Methods

### Study site description

#### Mombasa city

**Fig 1:**
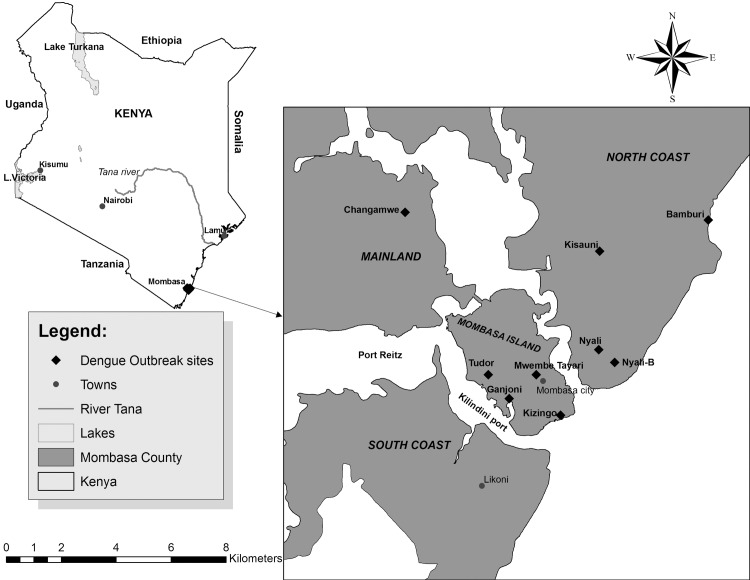
The overall map of Kenya and Mombasa city showing the dengue outbreak sites where vector sampling was conducted.

The dengue outbreak occurred in Mombasa, the second-largest coastal city in Kenya with a population of about 1.2 million [39]. It is situated at 4°0'S, 39°36'E and 5 m (18 ft) above sea level; and is characterized by a flat topography [40]. It consists of Mombasa Island which is separated from the mainland by Tudor Creek and Kilindini Harbour. The island is also connected to the northern mainland (North Coast) by the Nyali Bridge, to the south (South Coast) by the Likoni Creek and to the west by the Makupa Causeway. There are several prime (Kizingo) and middle class (Ganjoni and Tudor) residential areas on the island as well as several schools and colleges. In the North Coast is Nyali, a prime up-market residential area, with the most prestigious academic institutions. Bamburi, an outlying township along the Mombasa-Malindi road is also situated here. In the South Coast is Likoni, a lower income and lower-middle-class neighborhood while on the mainland along the Mombasa-Nairobi Highway lies Changamwe which is an industrial area with low-class housing estates (Fig 1).

#### Weather data

**Fig 2:**
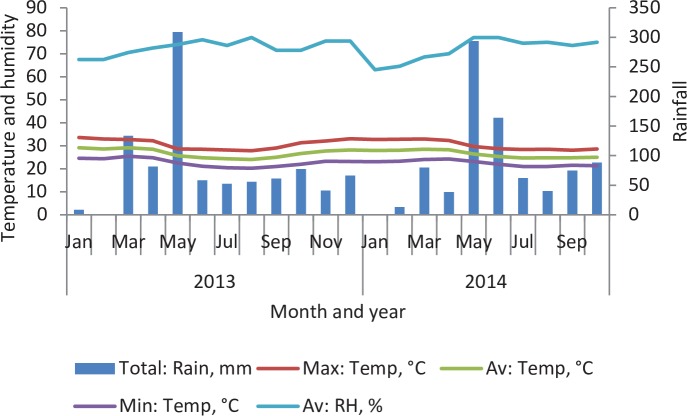
Rainfall, temperature and relative humidity prevailing in Mombasa at the time of mosquito sampling in 2013 and 2014 (Source: Kenya Meteorological Department, 2013 and 2014).

Generally, Mombasa city experiences a tropical wet and dry climate [40]. The amount of rainfall is dependent on the season with April and May (long rain season) receiving the highest amount, November to December (short rain season) moderate amount while January to February is the driest and receives minimal amount of rainfall. The total annual precipitation averages 1072.7 mm, while the mean annual temperature is 26.3°C. During the outbreaks, temperatures were relatively high especially November to December 2013. Weather data for Mombasa during the outbreaks were obtained from the Kenya Meteorological Department (Fig 2).

#### Epidemiological data

Two outbreak peaks occurred from April to June and November to December in 2013, and one from March to June in 2014. While the April to June 2013 and March to June 2014 outbreaks occurred in the larger Mombasa city, the November to December 2013 was localized only at Nyali-B (a small unique government institution in Nyali area). According to the institution’s medical doctor, approximately 36% of the total population was affected, 17% of whom were hospitalized.

### Study design and site selection

Entomological “outbreak” investigations were launched as a result of detected dengue transmission in humans in Mombasa city. Sampling locations were selected purposely based on the occurrence of laboratory-confirmed dengue cases. Within the locations, specific sites and households were selected randomly. Due to limited resources, investigations were conducted for a short period of time in only 7 out of the 9 affected locations.

### Entomological surveillance

Entomological sampling was conducted from 21 to 28 April 2013 and 28 November to 2 December 2013, while the 2014 sampling was from 4 to 15 March. Mosquitoes were collected indoors and outdoors, as larvae and adults, on a daily basis for the duration of each visit. One of the challenges of indoor sampling was the extreme difficulty in accessing some of the residences, especially in more affluent areas such as Nyali and Kizingo as the residents insisted on preserving their privacy.

### Adult mosquito collection

Several sampling tools were employed to capture adult *Ae*. *aegypti*. Ten BG-Sentinel traps (Biogent), the current gold standard for adult *Ae*. *aegypti* surveillance, were set outside houses and monitored daily for three consecutive days in each site. Although resting boxes (RB) are not usual surveillance tools for *Ae*. *aegypti*, these devices were tested in Mombasa to determine their efficacy for possible future use. A total of six RBs were placed outside the same houses where BG-traps were deployed in Kizingo and Nyali and also monitored for three consecutive days. Electromechanical aspirators, which included backpack/Prokopack (BP/PP) aspirators, were used to collect indoor resting adult mosquitoes. The time spent at each house varied depending on the size and number of rooms. Additionally, 10 CO_2_-baited CDC light traps (LT), (John W. Hock Company, Gainesville, FL, U.S.A.) were hung at least two meters from the ground either immediately outside the houses or along the edges of the compound. Each trap was baited with 0.5 kg of dry ice held in igloos next to the traps [41] and left on site from dusk to dawn.

Mosquitoes were retrieved from the traps early every morning (and evening in the case of BG-Sentinel traps) and transported to a temporary site laboratory in Mombasa where they were knocked down using triethylamine (TEA). Collection of mosquitoes from indoors was conducted during the day using BP/PP aspirators. All collected mosquitoes were sorted, morphologically identified to species using keys [42–45] and pooled (≤ 25 mosquitoes per pool) by sex, species, collection method and date. All identification was done on ice packs to preserve the virus for isolation work in cell culture. Identified mosquitoes were preserved in 1.5-ml cryogenic vials and transported in liquid nitrogen to the biosafety level 2 Arbovirus and Viral Hemorrhagic Fever (VHF) Laboratory at KEMRI for analysis.

### Immature *Ae*. *aegypti* collections

All water-holding containers found indoors (inside every accessible house) and outdoors (outside the houses and within the peridomestic environment) including some natural habitats such as tree holes and plant leaf axils were inspected using flashlights where necessary. Samples from each positive container were collected using ladles and pipettes or, in the case of jerry cans, the water was poured through a sieve onto a white basin and the larvae or pupae then picked from the sieve using Pasture pipettes. The samples were linked by geo-coding using a GPS to the premises where they were collected. Live immature mosquitoes sampled from each water container type were reared to adults and identified to species as for adult collections. Indoor and outdoor containers were then scored separately as either being wet negative (with no *Ae*. *aegypti* immatures) and wet positive (with at least one immature *Ae*. *aegypti*), were then scored separately.

### Larval indices

The mosquito indices were calculated as House Index (HI)—the percentage of houses positive with immature mosquitoes, Container Index (CI)—the percentage of water holding containers in which mosquito breeding is occurring and Breteau Index (BI)—the number of positive containers per 100 houses [46]. The following formulas were used to determine these indices:

$$\mathrm{HI}=\frac{\mathrm{Number of houses with immature mosquitoes}}{\mathrm{Number of inspected houses}}\times 100$$

$$\mathrm{CI}=\frac{\mathrm{Number of containers with immature mosquitoes}}{\mathrm{Number of wet containers}}\;\text{x}\;100$$

$$\mathrm{BI}=\frac{\mathrm{Number of containers with immature mosquitoes}}{\mathrm{Number of inspected houses}}\;\text{x}\;100$$

### Mosquito processing and virus isolation in cell culture

Mosquito pools were homogenized in a biosafety level 2 laboratory at KEMRI’s Centre for Virus Research using 4.5-mm diameter copper beads (BB-caliber airgun shot) in 1 ml of Minimum Essential Medium Eagle (MEM), with Earle’s salts and reduced NaHCO3 (Sigma-Aldrich, St. Louis, MO) supplemented with 15% heat-inactivated fetal bovine serum (FBS; Sigma-Aldrich), 2% L-glutamine (Sigma-Aldrich), and 2% antibiotic/antimycotic solution (Sigma-Aldrich) with 10,000 U penicillin, 10 mg streptomycin, and 25 μg amphotericin B per milliliter. The homogenates were clarified by centrifugation at 12000 rpm (Eppendorf centrifuge 5417R) for 10 min at 4°C and the supernatants transferred into 1.5-ml cryogenic vials. Each mosquito pool supernatant (50 μl) was inoculated in a single well of a 24-well culture plate containing a confluent monolayers of Vero cells (CCL81) grown in MEM, which was supplemented with 10% FBS and 2% L-Gulatamine and 2% antibiotic/antimycotic solution. The inoculated cultures were incubated for 45 min to allow for virus adsorption, and each sample maintained in MEM supplemented with 2% FBS and 2% antibiotic/antimycotic solution. The cultures were incubated at 37°C in 5% CO_2_ and monitored daily, through day 14, for cytopathic effects (CPE) as an indication of virus infection. The samples were also inoculated in C6/36 *Aedes albopictus* cells) grown in Dulbecco’s Modified Eagle’s Medium (DMEM) (Sigma-Aldrich) and incubated at 28°C.

### Total RNA isolation and virus identification by reverse transcription PCR

Total RNA was isolated from the supernatant of each *Ae*. *aegypti* mosquito pool and culture exhibiting CPE by the Trizol-LS-Chloroform method [47]. Extracted RNA was reverse transcribed to cDNA using SuperScriptIII reverse transcriptase (Invitrogen, Carlsbad, CA) and random hexamers followed by RT-PCR using AmpliTag Gold PCR Master Mix (Applied Biosystems) [48]. The cDNA was tested using a panel of general (*alphavirus* and *flavivirus*) and consensus primers for DENV [49–51]. A positive control cDNA and a no-template negative control were included during the setting up of all PCR reactions. Amplification products were resolved in 1.5% agarose gel in Tris-Borate-EDTA buffer stained with ethidium bromide.

## Results

### Container type and positivity for *Ae*. *aegypti* immatures

An overall total of 1,676 containers were inspected indoors and outdoors. From these, jerry cans were the most abundant, 704 (42%), followed by tires, 242 (14%), plastic buckets, 228 (14%) and drums, 169 (10%). However, tires had the highest percentage of *Ae*. *aegypti* larvae/ pupae positivity, 165 (68%) by container type among the most sampled containers, followed by drums, 71 (42%), plastic buckets, 64 (28%) and jerry cans, 106 (15%).

### Indoor container types and positivity for *Ae*. *aegypti* immatures

A total of 827 containers were sampled indoors and 158 of them found positive for *Ae*. *aegypti* immatures, giving an indoor CI of 19. Indoor containers were also less diverse (7 container types) and although jerry cans were the most abundant (61%) only 12% of them were positive while 39% each of drums and plastic basins were positive. No immatures were sampled in clay pots and plastic bottles (Table 1).

**Table 1:** Rank distribution of wet containers sampled indoors and immature *Ae*. *aegypti* positivity by container type in Mombasa city during the outbreaks.

Container type	Number (% of total)	Rank	Wet–ve ^a^ (%)	Wet +ve ^b^ (%)
Jerry cans	508 (61)	1	446 (88)	62 (12)
Plastic buckets	145 (18)	2	114 (79)	31 (21)
Drums* (plastic, metal)	124 (15)	3	77 (61)	47 (39)
Plastic basins	38 (5)	4	23 (61)	15 (39)
Flower pots	5 (<1)	5	2 (40)	3 (60)
Clay pots	4 (<1)	6	4 (100)	0 (0)
Plastic bottles	3 (<1)	7	3 (100)	0 (0)
Total	827 (100)		669 (81)	158 (19)

^a^Number of wet containers with no *Ae*. *aegypti* immatures

^b^ Number of wet containers with at least one immature *Ae*. *aegypti*

*50–200 liter capacity; +ve = positive; -ve = negative

### Outdoor container types and positivity for *Ae*. *aegypti* immatures

A total of 849 containers were sampled outdoors and 362 of them found positive for *Ae*. *aegypti* immatures, representing an outdoor CI of 43%. Outdoor containers were also more diverse (35 container types). Tires were the most abundant, 242 (29%) and most positive by container type (68%). These were followed by jerry cans, 196 (23%) of which only 22% were positive (Table 2).

**Table 2:** Rank distribution of wet containers sampled outdoors and immature *Ae*. *aegypti* positivity by container type in Mombasa city during the outbreaks.

Container type	Number (% of total)	Rank	Wet -ve^a^	Wet +ve^b^ (%)
Tires	242 (29)	1	77	165 (68)
Jerry cans	196 (23)	2	152	44 (22)
Plastic buckets	83 (10)	3	50	33 (40)
Broken bottles	60 (7)	4	50	10 (17)
Tanks^†^	54 (6)	5	23	31 (57)
Clay pots	49 (5)	6	42	7 (14)
Drum* (plastic, metal)	45 (2)	7	21	24 (55)
Plastic basins	20 (2)	8	12	8 (40)
Metal cans	19 (2)	9	12	7 (37)
Solar heaters	16 (2)	10	10	6 (38)
Flower pots	12 (1)	11	9	3 (25)
Plastic bottles	9 (1)	12	7	2 (22)
Plates (plastic, metal)	4 (<1)	13	2	2 (50)
Plastic chairs	4 (<1)	14	2	2 (50)
Glass jars	3 (<1)	15	1	2 (67)
Natural containers (Banana axils, coconut shell)	3 (<1)	16	1	2 (67)
Trays (plastic, metal)	3 (<1)	17	2	1 (33)
Others^§^	27 (3)	18	14	13 (48)
Total	849 (100)		487 (57)	362 (43)

^a^ Number of wet containers with no *Ae*. *aegypti* immatures

^b^ Number of wet containers with at least one immature *Ae*. *aegypti*

*50–200 liter capacity

^†^≥500 liter capacity

+ve = positive; -ve = negative

^§^ (Included animal watering pan, blocked manhole, car bumper, ceramic cup, ceramic pot, discarded bathtubs, discarded car, discarded kitchen sinks, discarded toilets, fish pond, gulley trap, plastic bucket lid, polythene bag, Styrofoam boxes, swimming pool, water fountains).

### House, Container and Breteau indices

An overall total of 192 houses was sampled (between 3 and 70 per site) of which 42 were positive for *Ae*. *aegypti* immatures, representing a HI of 22%. A total of 1,676 containers was also inspected indoors and outdoors and 520 (31%) were positive, with an overall CI of 31% and BI of 270.1. All these indices exceeded the WHO-documented thresholds for risk of dengue outbreak/transmission: all indices >1, HI > 1% and BI > 5, suggesting that all the areas sampled were at risk of dengue transmission (Table 3).

**Table 3:** Overall and site-specific House, Container and Breteau indices for Mombasa city during the outbreak.

Sampled areas	No. of houses sampled	No. of houses +ve for immatures	HI	No. of containers with water	No. of containers +ve for immatures	CI	BI
Bamburi	13	3	23.1	44	16	36.4%	123.1
Changamwe	70	16	22.9	832	251	30.2%	358.6
Ganjoni	3	2	66.7	14	5	35.7%	166.7
Kizingo	29	3	10.3	395	92	23.3%	317.2
Nyali-B*	42	16	38.1	185	69	37.3%	164.3
Nyali	15	1	6.7	70	27	38.6%	180.0
Tudor	20	1	5.0	136	60	44.1%	300.0
Total	192	42	21.9	1676	520	31.0%	270.1

BI = Breteau index; CI = Container index; HI = House index

*All the houses in Nyali-B were sampled

No. = number; +ve = positive

### Container type and productivity for *Ae*. *aegypty* immatures by site

From the overall 520 positive containers, 2,510 *Ae*. *aegypti* immatures emerged into adult mosquitoes, of which 76% were from large containers: jerry cans, 451 (18%), drums, 431 (17%), buckets, 404 (16%), tires, 359 (14%) and large water tanks, 253 (10%). Although jerry cans were the most productive containers overall, each site had specific container types that were most productive. For instance, tanks and drums were the most and second most productive containers in Kizingo, with 17% and 16%, respectively. Drums were the most productive in Nyali (30%) and Changamwe (33%), plastic basins (35%) in Nyali-B, tires (82%) in Bamburi, plastic buckets (30%) and jerry cans (30%) in Tudor while plastic buckets (81%) were the most productive in Ganjoni (Table 4).

**Table 4:** Overall rank distribution of immature *Ae*. *aegypti* totals by site and container type in Mombasa city during the outbreak.

Total *Ae*. *aegypti* immatures by container type and site																						
	Kizingo			Nyali			Nyali-B			Bamburi			Tudor			Changamwe			Ganjoni			
Container type	F	M	Total	F	M	Total	F	M	Total	F	M	Total	F	M	Total	F	M	Total	F	M	Total	Overall total
Jerry cans	49	113	162	4	13	17	6	7	13	0	0	0	65	59	124	60	56	116	19	0	19	451
Drums* (plastic, metal)	95	87	182	11	12	23	18	28	46	0	0	0	15	33	48	72	60	132	0	0	0	431
Plastic buckets	37	49	86	0	0	0	60	46	106	2	2	4	76	50	126	0	0	0	77	5	82	404
Tires	77	70	147	0	0	0	0	0	0	27	29	56	24	44	68	44	44	88	0	0	0	359
Tanks^†^	108	88	196	0	0	0	0	0	0	0	0	0	23	32	55	2	0	2	0	0	0	253
Plastic bottles	39	35	74	0	0	0	0	0	0	0	0	0	0	0	0	53	12	65	0	0	0	139
Plastic basins	0	0	0	0	0	0	70	57	127	2	6	8	0	0	0	0	0	0	0	0	0	135
Polythene bags	52	71	123	0	0	0	0	0	0	0	0	0	0	0	0	0	0	0	0	0	0	123
Cans	40	36	76	9	5	14	0	0	0	0	0	0	0	0	0	0	0	0	0	0	0	90
Discarded kitchen sinks	15	20	35	0	0	0	0	0	0	0	0	0	0	0	0	0	0	0	0	0	0	35
Old bath tabs	17	16	33	0	0	0	0	0	0	0	0	0	0	0	0	0	0	0	0	0	0	33
Broken glass jars	18	10	28	0	0	0	0	0	0	0	0	0	0	0	0	0	0	0	0	0	0	28
Water fountains	0	0	0	13	0	13	0	0	0	0	0	0	0	0	0	0	0	0	0	0	0	13
Others^§^	4	2	6	5	5	10	0	0	0	0	0	0	0	0	0	0	0	0	0	0	0	16
Total	551	597	1,148	42	35	77	154	138	292	31	37	68	203	218	421	231	172	403	96	5	101	2,510

F = female; M = male

*50–200 liter capacity

^†^≥500 liter capacity

^§^(animal watering pan, blocked manhole, car bumper, ceramic cup, ceramic pot, discarded bathtubs, discarded car, discarded kitchen sinks, discarded toilets, fish pond, gully trap, plastic bucket lid, polythene bag, Styrofoam boxes, swimming pool, water fountains).

### Immature *Ae*. *aegypti* collections indoors and outdoors by site

Of the 2,510 *Ae*. *aegypti* immatures collected over the entire sampling period, 995 (40%) were from indoors and 1,515 (60%) from outdoors. Kizingo recorded the highest number (1,148) especially outdoors while Nyali (77) and Bamburi (68) recorded the least (Table 5)

**Table 5:** Number of *Ae*. *aegypti* immatures collected indoors and outdoors by site.

	Indoor			Outdoor		
Site	F	M	Total	F	M	Total
Changamwe	142	107	249	89	65	154
Ganjoni	94	5	99	2	0	2
Kizingo	124	93	217	427	504	931
Tudor	39	36	75	164	182	346
Nyali	20	17	37	22	18	40
Nyali-B	154	138	292	0	0	0
Bamburi	9	17	26	22	20	42
**Total**	582	413	**995**	726	789	**1,515**

F = females; M = males

### Mosquitoes collected as adults

A total of 5,461 adult mosquitoes of diverse species were collected indoors and outdoors by a combination of methods. The majority of mosquitoes collected were *Cx*. *pipiens*, 2,979 (55%) followed by *Ae*. *aegypti*, 2,065 (38%), (Table 6).

**Table 6:** Mosquito species collected as adults by various methods combined.

	No. collected (No. of pools)			
Species	Females	Males	Total (pools)	% of total
*Aedes simpsoni*	1(1)	0(0)	1(1)	<0.1
*Ae*. *tricholabis*	74(3)	0(0)	74(3)	1.4
*Ae*. *vittatus*	3(3)	1(1)	4(4)	<0.1
*Ae*. *aegypti*	1,001(190)	1,064(163*)	2,065(353)	37.8
*Aedeomyia furfurea*	2(2)	0(0)	2(2)	<0.1
*Culex univittatus*	6(2)	0(0)	6(2)	<0.1
*Cx*. *vansomereni*	13(7)	0(0)	13(7)	0.3
*Cx*. *zombaensis*	29(6)	44(5)	73(11)	1.3
*Cx*. *pipiens*	911(163)	2,068(224)	2,979(387)	54.5
Others	n/a	n/a	244(32)	4.5
Total	2,040(311)	3,177(393)	5,461(737)	100

na = not applicable; No. = Number

* One pool of male mosquitoes was positive for DENV

### Adult *Ae*. *aegypti* collections indoors and outdoors by various sampling tools

Only 78 (4%) adult *Ae*. *aegypti* mosquitoes were collected indoors while the majority, 1,987 (96%), were from outdoors. Overall, most of the collections were from Mwembe Tayari, 521 (25%), Kizingo, 317 (15%) and Ganjoni, 312 (15%), while Bamburi recorded the least, 21 (1%). The BGS traps, which were used in all the sampling periods, collected the highest number, 1,460 (73%) followed by CDC light traps, 347 (18%) while resting boxes, used only once during the April 2013 collection, yielded the least, 11 (1%).

### Dengue virus isolation

Out of 273 pools of *Ae*. *aeypti* sampled as immatures and reared to adults and those sampled as adults, identified and processed, one DENV-2 was isolated in Vero cells, and confirmed by RT-PCR [49], from a pool of 2 male mosquitoes collected as adults, representing a minimum infection rate (MIR) of 0.2. No DENV was detected in pools of *Ae*. *aegypti* homogenates directly by RT-PCR.

## Discussion

The April-June 2013 and March-June 2014 dengue outbreaks coincided with the long rain seasons along the coast of Kenya. These rains may have resulted in increased aquatic habitats for *Ae*. *aegypti* breeding [52], thus increasing the vector population density and the risk of dengue transmission. However drought also promotes vector abundance through increased storage of water in which *Ae*. *aegypti* mosquitoes breed [53]. For example the isolated outbreak that was reported in Nyali-B occurred at a time of diminished rainfall reported to be less than 50 mm per month by the Kenya Meteorological Department. Nyali-B, a government institution with dormitories housing approximately 150 people, had no piped water at the time of the outbreak and the residents were storing water in many open container types indoors. Outdoor water storage containers were comprised mainly of large water tanks that were difficult to sample from hence the observed low frequency of *Ae*. *aegypti* immatures collected outdoors relative to indoors. The water storage practices resulted in high CI, HI and BI of 37%, 38% and 164.3, respectively, for the Nyali-B dormitories. Overall, the CI of 31% (indoor, 19%; outdoor, 43%), HI (21.9) and BI (270.1) observed for Mombasa in general were also high and well above the WHO-documented thresholds, suggesting that most of the areas sampled in Mombasa city were at risk of dengue transmission. However these threshold levels are controversial since transmission can still occur even when the indices are safely low or fail to occur even when they are high [54,55]. The thresholds also differ from place to place [37] and are affected by human serotype-specific herd immunity and ambient temperature [56]. Hence pupal indices have been recommended instead as the most appropriate for assessing DENV transmission risk especially since there is also no correlation between larval indices and actual pupae that emerge to contribute to adult population [36,57].

Out of all mosquito species collected, only *Ae*. *aegypti* is known to transmit DENV in urban areas. *Ae*. *vittatus* and *Ae*. *simpsoni* both of which co-exist with *Ae*. *aegypti* [2] while the *Culex* spp. have not been associated with DENV. The high number of *Ae*. *aegypti* caught implies that the dengue vector is well established in Mombasa and the risk of DF, chikungunya and yellow fever transmission is high in the absence of effective vector control. Adult *Ae*. *aegypti* mosquitoes collected indoors were fewer than those collected outdoors, probably reflecting different capture efforts and techniques. However the large number of immatures collected indoors suggests that the coastal *Ae*. *aegypti* population breeds indoors just as successfully as outdoors, subject to availability of aquatic habitats, but is mostly an outdoor resting mosquito. Previous studies in Rabai, also a coastal town, demonstrated differential domesticity of *Ae*. *aegypti* [59]

Kizingo, one of the most affluent regions, recorded the highest number of *Ae*. *aegypti* immature collection in April 2013 and this is attributed to the heavy construction work that was on-going at the time of the outbreak. The many containers which were serving as water reservoirs for the construction work may have provided favorable aquatic habitats for the mosquitoes. Therefore, construction sites should be monitored closely as important sources of *Ae*. *aegypti* mosquitoes and especially targeted for vector control to reduce the risk of dengue transmission. Dengue cases were also reported in areas which had low vector densities. This is in agreement with previous observation that low vector density does not always result in lower levels of dengue transmission because a single infected mosquito can transmit the virus to many people given its day biting, anthropophilic, interrupted and multiple-biting behavior [60].

This study has demonstrated further the efficacy of BGS traps which collected 73% of all adult *Ae*. *aegypti* followed by the CO_2_-baited CDC light traps (18%) and BP/PP aspiration (9%) respectively. While BGS traps were only used outdoors during all sampling periods, the BP/PP devices were used mostly to collect resting mosquitoes indoors in only the houses that we were permitted to enter, and only twice out of the three sampling occasions, a situation that may have influenced the catch. Previous studies have also found the BG-Sentinel traps comparatively more effective than other tools [61]. The relatively large number of *Ae*. *aegypti* mosquitoes collected by the CO_2_-baited CDC light traps suggests that this tool can significantly complement other tools in the surveillance of *Ae*. *aegypti* at the coastal region of Kenya. While it is difficult to understand how a day-feeding mosquito species can be attracted to CO_2_ at night, it is possible that *Ae*. *aegypti* starts to seek blood meals very early in the morning, perhaps before the traps are collected or they feed beyond sundown allowing some to be attracted to light traps.

In general, this study demonstrated a relationship between the number of containers, level of positivity and adult productivity. For example, the most sampled containers were jerry cans, discarded tires plastic buckets and drums, an observation similar to that made in previous studies in Malindi district, also in coastal Kenya, where these same containers were the highest in positivity and the most productive, although not in the same order [62]. However, container productivity varied by site depending on the type of containers commonly used for water storage regardless of the social status.

Establishment of a disease in an area depends on a number of factors including its maintenance mechanism including appropriate competent vector, availability of amplifying hosts and favorable climatic conditions. Isolation of DENV-2 from a pool of male *Ae*. *aegypti* mosquitoes collected as adults during this study marks the first such case in Kenya and provides further evidence that DENV may have maintenance mechanisms that include vertical transmission by mosquitoes [63,64]. This may have epidemiological significance with regard to the maintenance of DENV in nature in conditions adversarial to the virus. The coastal region in Kenya is usually characterized by high temperatures, throughout the year, that favor the proliferation of DENV and subsequent transmission by *Ae*. *aegypti* [65,66]. This likely explains the widespread occurrence of dengue cases across the city this time [22].

Considering these factors, dengue fever will likely be a recurrent problem at this coastal city going forward. We recommend that organized vector surveillance and control programs against *Ae*. *aegypti* mosquitoes be instituted in Mombasa, in particular, and in Kenyan in general, where currently vector control activities focus on malaria vectors only, as in other parts of Africa [67]. Vector control should involve public participation to focus on routine clean-up campaigns to reduce mosquito-producing containers, a basic step to prevent and/or control dengue outbreaks. This activity should target all container types with the potential to hold water, since this study has demonstrated that the dengue vector can successfully breed in a wide range of container types, and also construction sites for targeted source reduction and maximum adult reductions [54,68]. Community participation through sensitization, public awareness about the disease and the best practices of preserving water and disposal of tires and containers would be key in reducing *Ae*. *aegypti* densities. Also providing a reliable supply of piped water in every household would reduce the need for water storage containers that also act as aquatic habitats for dengue vectors. However, success of these efforts will require legislation and proper inter-agency (health and environment) coordination and funding, with the support of the national and county governments. In addition, training of vector control personnel on *Ae*. *aegypti* biology, surveillance and control based on the WHO guidelines [10] should be prioritized.
